# Two Generations of Lunacy Legislation

**Published:** 1919-03-01

**Authors:** 


### TWO GENERATIONS OF LUNACY LEGISLATION.

### Its Absurdities and Reform.

Finality in Legislation, especially in Lunacy Legislation, is not to be attained. For the last two generations there has been an almost constant clamour for new Lunacy Legislation, and many new Lunacy Laws have been passed. The Lunacy Act, 1890, was intended and expected to be a permanent enactment. In less than thirty years it is completely out of date, and the basis on which it was founded is obsolete. All Lunacy Legislation hitherto has been founded primarily on the principle of safeguarding the liberty of the subject; but nobody now cares anything about the liberty of the subject. Patients who are sent to Lunatic Asylums are sent under a magistrate's order, and cannot be otherwise received. But patients who are sent equally against their wills to a fever hospital are not sent under a magistrate's order, nor are any legal formalities required. Yet no person's life or health is endangered by being sent to a Lunatic Asylum, while the danger to both life and health in a fever hospital is always present and cannot be avoided. Yet there is no clamour for a magistrate's order to authorise the reception and detention in a fever hospital of a patient suffering from smallpox or scarlet fever. Workmen habitually and constantly interfere with the liberty of their fellows to work what hours they please, for what employers they please, and to turn out as much work as they please. The liberty of any panel patient to choose his own doctor is interfered with; the liberty of a cow-keeper to keep his cows as he pleases, and to milk them as he pleases is constantly interfered with; and in every form of human endeavour the liberty of every man is constantly being interfered with in a hundred different directions. The liberty of the subject is become an exploded superstition, and nobody cares one straw about it.

On the other hand, a new notion is arising with respect to the treatment of the insane. It is becoming recognised that they are being received and detained in institutions not solely to safeguard society from the mischief that may result from their being at large, but also that they themselves may receive treatment with a view to their being restored to health, and taking their places again in the world. It is recognised that a large proportion of those who suffer from madness do in fact recover, and it is recognised that every effort possible should be made to increase this proportion, and to diminish the duration of their illness. This is a new conception, and it is upon this that the Lunacy Legislation of the future is to be founded.

For this purpose it is necessary that the reception of lunatics in asylums should be facilitated instead of being obstructed. At the present time the removal of a mad person to a lunatic asylum is hedged about with so many formalities and restrictions that the removal is always delayed, and is sometimes altogether prevented. The object of future legislation must be to get the lunatic under care as soon as possible, and with as little friction and as little formality as possible.

There is a prejudice against lunatic asylums, and against calling mad persons lunatics or madmen or insane persons. The prejudice is unreasonable. The fact is not altered by attaching a name to it, and disgrace, if any, attaches not to the name, but to the fact, and cannot be altered by alteration of the name as long as the fact exists. Lunacy authorities and Lunacy doctors who ought to know better constantly confuse the name with the fact, and consider that if they alter the name, they will alter the thing. The notion is childish and contemptible, and the effort of reformers should be addressed not to altering names but to altering procedure, to altering the childish prejudice that exists in the minds of the public against calling a spade a spade, and a madman a madman.

The alienists or mad-doctors, by whatever name they call themselves, are unanimous in declaring that the earlier a mad person is placed under treatment the better is his chance of recovery. The declaration is misleading. There is practically no treatment for insanity, except that of removing the patient from home, and into different surroundings. That is the whole of the treatment. In these new surroundings he is taken care of, he is well fed, he is safeguarded from the results of his own conduct, he is amused, and in some institutions, and for some of their inmates, occupation is found for him. If this be called treatment, then the lunatic is treated, but of such treatment as is ordinarily termed medical there is none. Still, under these conditions a considerable proportion of patients afflicted with madness do recover, and begin to recover when they are removed from their own homes, and placed under these conditions. It is eminently desirable, therefore, that removal from home into these conditions should be in every way facilitated, and that all obstacles to the removal should be abolished. This must be the aim of future legislation, and it is a gratifying sign of the times to find that the Conference of Asylum Visiting Committees is alive to its desirability.

There are various ways in which this important object can be facilitated. The absurd formalities which stand in the way of a patient entering an institution as a voluntary boarder should be abolished. Permission should be given for voluntary boarders to be received into public asylums as well as into registered hospitals and licensed houses. The magistrate's order for the reception of a lunatic into an institution or into private care should be abolished. An out-patient department for mental diseases should be added to every general hospital. Experience shows that such departments in the few hospitals in which they have been established are well frequented, and patients who find themselves suffering from mental disease are willing and anxious to avail themselves of the advice that th

**Lunacy Legislation—(*continued*).**

receive at such places. If such an out-patients' department were established in connection with every general hospital, the need of early advice in all cases of mental disease among the poor would be met without the establishment of special clinics in special places for the treatment of mental disease alone.

The main difficulty would be not the provision of buildings, for plenty of them already exist, but the provision of medical men skilled in the treatment of mental disease. Of the cases of mental disease that present themselves at an out-patient clinic only a very small proportion are cases of madness, and for the treatment of mental disease that is not madness there is not, at present, any personnel available; but the demand would in time ensure a supply.

